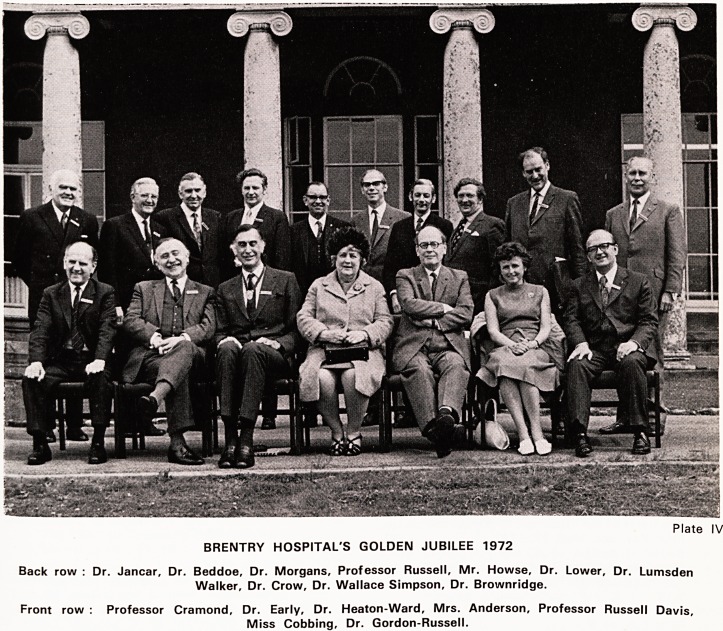# Fifty Years of Brentry Hospital
*Read at the first Spring Meeting of the South Western Regional Division of the Royal College of Psychiatrists, on Thursday, 13th April 1972, at Brentry Hospital, Westbury-on-Trym, Bristol, on the occasion of the celebration of the Hospital's Golden Jubilee.


**Published:** 1972-07

**Authors:** J. Jancar

**Affiliations:** Consultant Psychiatrist, Stoke Park Hospital Group. Lately Consultant Psychiatrist, Hortham-Brentry Group, Bristol


### Fifty Years of Brentry Hospital*
### (1922 - 1972)

**J. Jancar, M.B., B.Ch., B.A.O., F.R.C.Psych., D.P.M.**
Consultant Psychiatrist, Stoke Park Hospital Group.
Lately Consultant Psychiatrist, Hortham-Brentry Group, Bristol.

*"The sands of time are sinking year by year for all of us, but the memory of the great ones in the past is a spur to further effort, an example for a truer and higher life in us all. Happy shall we be if we can pass to our successors some of the professional honour and scientific attainment which has been handed down to us from our predecessors".*

Long Fox of Bristol 1901

### INTRODUCTION

To appreciate and to understand better the history and development of Brentry Hospital, whose Golden Jubilee we are celebrating this year, we must take a quick glance into the distant and the more recent history of some of the people and events which shaped the evolution of mental health in Bristol, a city which has played a prominent, and sometimes an original part in psychiatry. Its climate of opinion has been particularly favourable to new ideas and, from the 17th century to the present time, Bristol has given the pioneers practical support and encouragement in their work.

It was John Carey, a Bristolian, who, in 1696, founded St. Peter's Hospital. This was the first hospital where "pauper lunatics" received treatment and care and Dr. T. Dover was its first consultant physician. He is also remembered for his "Dover's powder" (pulvis ipecacuanhae et opii). During the next century various physicians from Bristol Royal Infirmary, instituted in 1735, held appointments at St. Peter's hospital, including the famous psychiatrists Dr. E. Long Fox, Senior, and Dr. J. C. Pritchard, who were also seeing psychiatric cases in the outpatient department at the Bristol Royal Infirmary.

At the time of the cholera epidemic in Bristol in 1832, some of the patients were transferred from St. Peter's Hospital to an old prison building at Stapleton, which had been built in 1779 to house Spanish, Dutch and French naval prisoners of war. Extensively rebuilt in 1861, it was renamed Stapleton Institution for Mental Defectives in 1918. In 1948, it became Stapleton Hospital for geriatric illnesses and was renamed Manor Park Hospital in 1956. In 1861, Fishponds Lunatic Asylum (now Glenside Hospital) was opened and 119 patients were transferred there from St. Peter's Hospital. Unfortunately, in 1940, St. Peter's Hospital was totally destroyed by enemy bombing. There was also a private lunatic asylum at Fishponds, opened in 1740 and managed by Dr. Joseph Mason.

The pioneering care of the mentally ill by "moral" treatment and group therapy was first introduced by Dr. E. L. Fox and his successor in a small Quaker Asylum at Cleeve Hill, Downend (about 1790), then in Brislington House (1804) and later in Northwoods Asylum, near Bristol. Dr. Henderson, the proprietor of Hanham House for Lunatics (about 1790) "governed his patients", according to John Wesley, "not by fear but love". In 1799, The Pneumatic Medical Institution was opened in 6/7 Dowry Square, Bristol, by Dr. T. Beddoes who was assisted by a young chemist, Humphrey Davy. The recorded observations on the release of emotion and other psychological phenomena by the inhalation of various gases, especially nitrous oxide, anticipated modern theories of drug abreaction in psychotherapy by more than a century. Later, Dr. Beddoes turned his attention to preventive psychiatry by early diagnosis.

Mary Carpenter (1807-1877) devoted her life to strays, unwanted and uncared-for children in Bristol. She founded reformatory and industrial schools in Bristol, among others the Kingswood Approved School, in 1852, and the Red Lodge Reformatory (1854). She was a pioneer in the treatment of juvenile delinquency (Saywell, 1964).

Stoke Park Colony was opened in 1909, with the appointment of Professor R. J. A. Berry as Director of Medical Services. In 1930, the first research centre for mental retardation was established there, and produced two brilliant research workers, Dr. R. M. Norman, neuropathologist, and Dr. J. A. Fraser Roberts, F.R.S., geneticist. When the Child Guidance Clinic was opened in Bristol in 1936 Dr. Ruth Griffiths, the psychologist from Stoke Park, assisted with the work there.

The Burden Neurological Institute was opened in 1939, under the directorship of Professor F. L. Golla. The Institute became nationally and internationally known for its work, especially for the study of electrophysiology of the central nervous system. Just before the second world war, the Burden Institute initiated the practice of electric convulsive therapy and, soon after, the first leucotomy in Great Britain was performed there.

The modern Barrow Mental Hospital was opened in 1938, and the Bristol Day Mental Hospital in 1951. The Bristol Industrial Therapy Organization was founded under the guidance of Dr. D. F. M. Early in 1960, to assist with the gainful employment of patients not fit for ordinary industry. In 1961, Dr. R. E. Hemphill organized, under the auspices of the Royal West of England Academy, the first exhibition to be held in Great Britain, which traced the evolution of mental health care, and illustrated the important contribution of Bristol in this field through nearly three centuries.

**Read at the first Spring Meeting of the South Western Regional Division of the Royal College of Psychiatrists, on Thursday, 13th April 1972, at Brentry Hospital, Westbury-on-Trym, Bristol, on the occasion of the celebration of the Hospital's Golden Jubilee.*

Bristol University, from its foundation in 1909, played an important role in the teaching of psychiatry and the importance of this subject was further recognised in 1962, when the Chair of Mental Health was added to the Faculty of Medicine and Dr. D. Russell Davies was appointed as the first Norah Cooke Hurle Professor of Mental Health.

### THE BURDENS

The Rev. Harold Nelson Burden and Mrs. Katherine Mary Burden came to Bristol in 1895, when Mr. Burden was appointed chaplain to Horfield Prison. Their arrival in Bristol opened a new chapter in the history of the care and treatment of down and outs and especially of mentally retarded people. Mr. and Mrs. Burden first came in contact with the people in real need and misery when they worked in the East End of London. They were also influenced by the work of Miss Octavia Hill (1842-1912), who was regarded as an authority on the lives of the poor in London and was a friend and disciple of Ruskin. Mrs. Burden worked for some time as her assistant. Mr. and Mrs. Burden were instrumental in the promotion and erection of the Royal Victoria Home near Horfield Prison. The home was, at first, intended for the care of inebriate women and girls in moral danger, but before it was completed a suggestion came from the Home Office that the work might, helpfully, also include the care of women convicts, whose crimes and history before and after conviction showed them to be suitable for the clemency of the Secretary of State. The suggestion was adopted and a wing was added for their reception.

The passing of the Inebriates Act of 1898, made much larger accommodation necessary for inebriates. Premises were therefore taken at Brentry (Plate I) and the assistance of County and County Borough Councils

Plate I — **Brentry House (about 1790).**

was sought. Twenty-four of these decided to contribute to the establishment of the Brentry Certified Inebriate Reformatory. Two villages were erected — the upper for females and the lower for males. Brentry House was adapted for officers and a residence for a superintendent. For three years, both Mr. and Mrs. Burden gave it their continuous attention. Every woman who entered the institution came under Mrs. Burden's influence and received her help (Clevedon Mercury 1919). Mr. Burden remained on the Brentry Certified Institution Board of Management as vice-chairman until his death in 1930.

A few years later, work for the mentally defective was added to the existing work of the National Institutions. They opened Stoke Park Colony in 1909, and gradually acquired more property. When Mrs. Burden died in 1930, there were over 2,000 beds at Stoke Park and ancillary hospitals. Mrs. R. Burden, the second wife of Mr. Burden, donated £10,000 in 1933, to establish the Burden Mental Research Trust for the study of the causation and inheritance of normal and abnormal mentality. She also gave financial support to the foundation of the Burden Neurological Institute in 1939. (Jancar, 1969). In 1969, The Burden Trust instituted an annual award — "The Burden Research Medal and Prize" for outstanding research work, to commemorate Mr. Burden, the foundation of Stoke Park Hospital and to encourage future research in the field of mental subnormality.

### BRENTRY CERTIFIED INSTITUTION

As the Inebriates Act proved to be ineffective and as the number of mentally defective patients was increasing, the Brentry Certified Inebriate Reformatory ceased to exist on the 3rd January 1922 when it became Brentry Certified Institution within the meaning of the Mental Deficiency Acts, 1913 and 1919. The newly designated institution provided 220 beds for male mentally defective patients over the age of 18 years. The aim of the institution was to occupy the patients as much as possible. The following industries were in operation soon after the establishment of the institution : General repairs to buildings, painting and decorating, carpentry, tailoring, boot making, basket making, mat making, sock making, wood bundling, farming and gardening. Education classes, sport and other social activities were provided by the staff and by funds from outside. There is an interesting excerpt from the annual report in 1923 by the Superintendent, Mr. Lambert, relating to the industrial therapy :

"The industrial improvements have already borne fruit and there is no doubt that by careful observation of, and as far as possible individual attention to, each case, a certain amount of useful work of some kind can be carried out by most of the patients, work that not only raises the morale but is conducive to physical fitness, often serves to mask or inhibit the mental perversion which so frequently exists, or to prevent its indulgence, and lastly and perhaps most important of all, serves to make the life of the defective a happier one".

Dr. H. L. Ormerod was appointed as the Medical Officer to the Institution. From 220 patients, 148 were from 17 contributing County Councils and 72 patients were from 45 various non-contributing Councils from England and Wales. In 1924, new workshops and a new infirmary were added to the existing buildings. Overcrowding was temporarily relieved with a new 60 bedded ward, "Warwick Ward', which was opened in 1927 to replace the old one which was burned down in 1925. The number of patients increased to 291.

In 1929, Dr. R. Fitzroy Jarret was appointed Resident Medical Superintendent of the Institution. The same year 500 books were purchased for the patients' library and wireless was of great interest and much appreciated by higher grade patients.

In 1930, Brentry Certified Institution was renamed Brentry Colony. To improve the standard of medical care of the patients and also to encourage research into the causes, treatment and prevention of mental retardation the following visiting consultants were appointed to Brentry Colony:

| | |
|---|---|
| Professor J. A. Nixon ...... | Physician |
| Mr. C. F. Walters ......... | Surgeon |
| Mr. J. Angell James ...... | Otolaryngologist |
| Mr. A. E. Iles ............... | Ophthalmologist |
| Dr. T. B. Wansbrough ... | Radiologist |
| Mr. G. Meadows ......... | Dental Surgeon |

The same year the Royal Medico-Psychological Association recognised the Colony as a Nurse Training School and a new concert hall and workshop were opened. Professor Nixon was very interested in the diet of the patients and his well remembered dictum was — good food is a first-class general sedative !

In 1931, Dr. Jarret took up a new post in Kent and Dr. G. de M. Rudolf (Plate II) was appointed the

Plate II

**Dr. G. de M. Rudolf, M.R.C.P., M.R.C.S., D.P.H., D.P.M.**

second Medical Superintendent. He continued the mental examination of all the patients, as started by Dr. Jarret and grouped the patients accordingly. He also increased the patients' recreational activities and encouraged more personal freedom and self-management of them. In 1935, a new hospital ward "Mather Jackson Hospital", named after the long-serving Chairman of the Board of Management, was opened which included a dispensary, medical and dental rooms. Dr. Rudolf resigned the post of Medical Superintendent in 1936, and was succeeded by Dr. J. J. Mason. Dr. Rudolf became visiting neurologist to the Colony until the beginning of the second world war, when he joined

Plate III

**Dr. J. F. Lyons, L.R.C.P. & S.I., D.P.H., D.P.M.**

the forces. After the war he became visiting psychologist and later consultant psychiatrist to Hortham/Brentry Hospital Group and Cambridge House (now Farleigh Hospital), until 1962 when he retired from hospital work.

The war years affected the progress of the Colony, but in spite of this and the depletion of staff, the morale and the patients' care remained exceptionally high. In 1948, Brentry Colony merged with Hortham Colony (which was built and opened in 1932 by the Bristol City authorities) forming the Hortham/Brentry Hospital Group.

Dr. J. F. Lyons (Plate III) became Group Medical Superintendent and Brentry Hospital continued to function as originally intended, to provide accommodation for adult male patients. Dr. W. A. Heaton-Ward took up his appointment as Deputy Medical Superintendent at Brentry Hospital in 1950. He remained there until 1954, when he became Medical Superintendent of Stoke Park Hospital Group. He introduced, among other things, rugby for the patients' recreation and their team competed successfully with Bristol rugby teams.

Dr. T. C. Leahy was appointed in 1955, as Senior Hospital Medical Officer to Brentry Hospital as successor to Dr. Heaton-Ward.

The same year, Dr. J. F. Lyons, Group Medical Superintendent and "father figure", highly respected and well loved by patients and staff alike, retired. Dr. W. L. Walker followed Dr. Lyons and held the post of Group Medical Superintendent with renewed vigour and new ideas, until 1966, when he left the group to take an appointment with United Bristol Hospitals. Dr. Grace E. Woods was Deputy Medical Superintendent from 1957 until 1964. In 1962, Dr. J. Jancar was appointed joint Consultant Psychiatrist to Stoke Park and Hortham/Brentry Groups with special responsibility to Brentry Hospital. He left Brentry Hospital in 1967, to be full time consultant to the Stoke Park Group and was succeeded by Dr. E. S. Lower. The Group had a further three consultant psychiatrists appointed: Dr. A. C. Fairburn in 1965, Dr. J. B. Gordon-Russell in 1967, and Dr. M. C. C. Bird in 1971. Apart from their other duties in the group and the community, they all have patients in Brentry Hospital.

During the past decade Brentry Hospital has witnessed and participated in the most exciting period of development in the field of mental subnormality, not only in Bristol but nationally and internationally. Numerous new discoveries, observations and schemes of multidisciplinary team approach opened the door to better treatment, care and rehabilitation and the prevention of mental and often associated physical retardation. Bristol's teaching and other hospitals, especially Southmead and Frenchay Hospitals, have given great support to the work in Brentry Hospital, Dr. F. J. W. Lewis undertaking chromosomal analysis and Dr. J. B. Holton biochemical examinations. They both have reported numerous abnormalities in patients in these fields. Dr. R. D. Eastham and his staff at Frenchay Hospital pathology department have been most helpful with special investigations of urine and blood which led to new observations and discoveries. These include raised plasma viscosity and low serum cholesterol in patients with Down's syndrome and other mentally retarded patients, and increased size of red cells in Downs' syndrome and epileptics and with advancing age in other patients. Professor L. S. Penrose, F.R.S., from London, was very helpful with the interpretation of dermatoglyphic patterns of patients' palm and foot prints. More recently Dr. T. J. David from the Bristol Royal Infirmary studied and reported anomalies in this field. (David, 1971).

The Burden Neurological Institute not only records and reports on E.E.G.'s of epileptics but also provides facilities and help with the research of other neurophysiological anomalies of patients. The clinical meeting this morning "Psychotherapy with Behaviourally Disturbed Young Men" illustrates this and also the team work of the hospital staff. Assessment of the patients in the Hospital and outside in the Assessment Clinic has been an established practice in Brentry Hospital for a long time (Jancar, 1971). Similarly the multidisciplinary case conferences and day care of the patients is a part of the hospital programme for better care and better community links.

The efficient nursing, administration and maintenance of Brentry Hospital has always been a great help to the well being of the patients and welfare of the staff. The dedicated and forward looking members of the Management Committee are the supporting influence in the running and further development of the services in the hospital. The hospital uses new techniques and the latest methods in dentistry, psychology, nursing, teaching, training, speech therapy, physiotherapy and other spheres to implement modern treatment and rehabilitation of patients.

Last year, two prefabricated wards were opened to relieve the overcrowding and two more are planned for this year.

**Chairmen of the Board of Management of Brentry Hospital :**

1922-1937 — Sir Henry Mather Jackson, Bt., C.B.E.
1937-1940 — W. Owen, Esq., J.P.
1940-1948 — G. Gibbs, Esq., J.P.

**Chairmen of the Management Committee of the Hortham/Brentry Hospital Group :**

1948-1951 — Professor J. A. Nixon, C.M.G., M.D., F.R.C.P.
1951-1952 — R. C. L. Fuller, Esq. (acting chairman)
1952-1964 — F. G. Jennison, Esq.
1964-to date Mrs. L. E. Anderson

### HOSPITAL LEAGUE OF FRIENDS

The League of Friends was formed in 1958, for the purpose of befriending patients without relatives and to assist in providing extra amenities beyond the resources of the Hospital Management Committee. They have provided, among many other things, an organ for the chapel, a patients' club room and a substantial sum of money towards the patients' swimming pool. The Managers of Brentry Hospital also are concerned with the religious life of the patients and they provided the hospital chapel. A part-time chaplain visits the hospital each week and takes services every Sunday. The patients of Brentry Hospital have been fortunate to receive much help and visits from various religious bodies and other voluntary organizations in the past and recent events have increased the interest of the public, and young people especially are coming forward to help.

### CONCLUSION

Fifty years of Brentry Hospital will, no doubt, leave a mark in the history of mental subnormality in Bristol, thanks to the great pioneering work and enthusiasm of medical, para-medical, nursing, administrative and other hospital staff, with the help they receive from so many people outside the hospital. It was a great honour and recognition of Brentry Hospital for the Royal College of Psychiatrists to hold its first spring meeting of the South Western Regional Division there during the celebration of the hospital's Golden Jubilee (Plate IV). It was rather fitting that the Chairman of the division, Dr. W. A. Heaton-Ward was a former Deputy Medical Superintendent of Brentry Hospital and that Dr. H. J. Crow, Clinical Director of the Burden Neurological Institute, who interprets E.E.Gs. for the hospital, should be the Honorary Secretary of the division.

Plate IV

**BRENTRY HOSPITAL'S GOLDEN JUBILEE 1972**

**Back row : Dr. Jancar, Dr. Beddoe, Dr. Morgans, Professor Russell, Mr. Howse, Dr. Lower, Dr. Lumsden Walker, Dr. Crow, Dr. Wallace Simpson, Dr. Brownridge.**

**Front row : Professor Cramond, Dr. Early, Dr. Heaton-Ward, Mrs. Anderson, Professor Russell Davis, Miss Cobbing, Dr. Gordon-Russell.**

The speakers at the Divisional Scientific Meeting were introduced by Professor D. Russell Davis, Professor of Mental Health and Dean of the Faculty of Medicine, University of Bristol.

1. The History of Brentry Hospital.

   Dr. J. Jancar, Consultant Psychiatrist, Stoke Park Hospital Group and Clinical Teacher in Mental Health, University of Bristol.

2. The Psychological Implications of Organ Transplantation.

   Professor William Cramond, Professor of Mental Health and Dean of the Medical School, University of Leicester.

3. Psychological and Perceptual Disorders in Anorexia and Obesity.

   Professor Gerald Russell, Professor of Psychiatry, Royal Free Hospital School of Medicine.

**ACKNOWLEDGEMENTS**

I am very grateful to Mr. R. W. G. Howse, Group Secretary, for making available the relevant hospital records and photographs; to Dr. H. Temple Phillips and Miss W. Wilcox for valuable historical information; to many members of the hospital staff for help with data and personal knowledge about the past history of the hospital and to Mrs. K. J. Hiscock and Mrs. Y. Wills for the secretarial work.

**REFERENCES**

1. Clevedon Mercury (1919) Nov. 8th. Katherine Mary Burden—50 years work: 1869 to 1919.
2. David, T. J. (1971) Dermatoglyphics in Medicine. Bristol med.-chir. J. **86,** 19.
3. Jancar, J. (1969) Sixty Years of Stoke Park Hospital (1909-1969). Bristol med.-chir. **J. 84,** 77.
4. Jancar, J. (1971) Assessment Unit for the Mentally Retarded. Bristol med.-chir. J. **86,** 27.
5. Saywell, Ruby J. (1964). Mary Carpenter of Bristol. F. Bailey & Son, Dursley, Glos.

## Figures and Tables

**Plate I f1:**
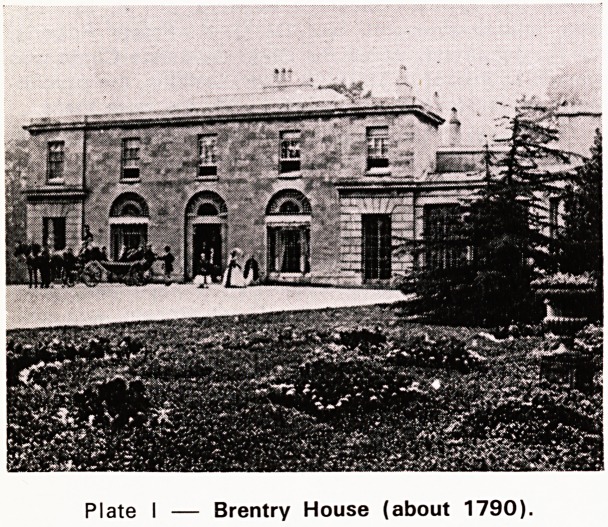


**Plate II f2:**
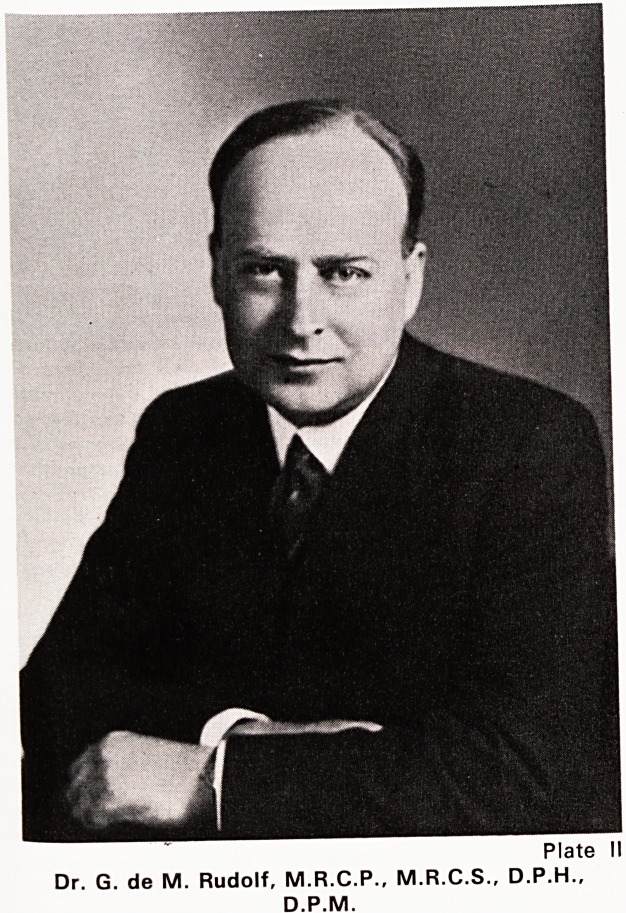


**Plate III f3:**
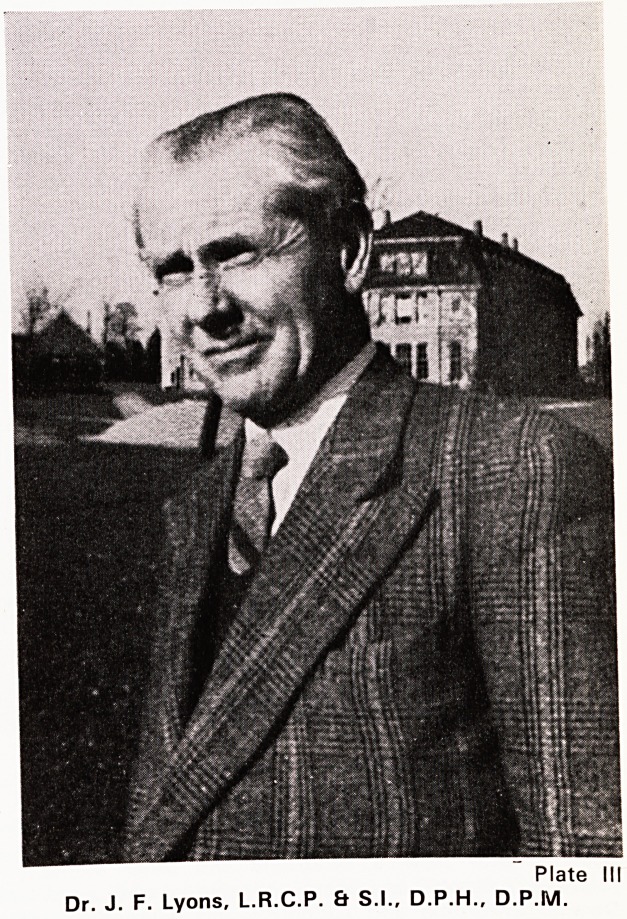


**Plate IV f4:**